# A randomized controlled trial adding behavioral counseling to supervised physical activity in people living with and beyond cancer (BOOST-UP-): a study protocol for a live remotely-delivered behavior change intervention

**DOI:** 10.1186/s12885-025-13904-8

**Published:** 2025-05-09

**Authors:** Linda Trinh, Ryan E. Rhodes, Shabbir M. H. Alibhai, Kristin L. Campbell, David M. Langelier, Eugene Chang, Tracey Colella, Brian Chan, Daniel Santa Mina, Paul Oh, Edward McAuley

**Affiliations:** 1 Faculty of Kinesiology and Physical Education, University of Toronto, 55 Harbord Street, Toronto, ON M5S 2W6 Canada; 2 School of Exercise Science, Physical and Health Education, University of Victoria, 11 Gabriola Rd, Victoria, British Columbia, V8P 5C2 Canada; 3 Department of Supportive Care, Princess Margaret Cancer Centre, 200 Elizabeth St, Toronto, ON M5G 2C4 Canada; 4 Faculty of Medicine, University of Toronto, 1 King’s College Circle, Medical Sciences Building, Toronto, ON M5S 1A8 Canada; 5 Department of Physical Therapy, University of British Columbia, 2177 Wesbrook Mall, Vancouver, BC V6T 1Z3 Canada; 6 Cummings School of Medicine, University of Calgary, 3330 Hospital Dr NW, Calgary, AB T2N 4Z5 Canada; 7 Multisystem and Musculoskeletal Rehabilitation Program, Toronto Rehabilitation Institute, University Health Network, 550 University Avenue, Toronto, ON M5G 2A2 Canada; 8 Toronto Rehabilitation Institute, KITE Research Institute, 550 University Avenue, Toronto, ON M5G 2A2 Canada; 9 Cardiovascular Rehabilitation Prevention and Rehabilitation Program, Toronto Rehabilitation Institute, 550 University Avenue, Toronto, ON M5G 2A2 Canada; 10 Department of Health and Kinesiology, University of Illinois at Urbana-Champaign, 906 South Goodwin Ave, Urbana, IL 61801 USA

**Keywords:** People living with and beyond cancer, Physical activity, Randomized controlled trial, Distance-based, Behavior change, Multi-process action control framework, Cancer survivors, Live remote

## Abstract

**Background:**

For many people living with and beyond cancer (LWBC), physical activity (PA) declines significantly after supervised PA interventions. The effect of short-term, supervised PA interventions on motivational outcomes and longer-term PA in people LWBC is limited, especially theoretically-based approaches to identify key motivational outcomes for behavior change. The purpose of this study is to compare the effects of a 6-month virtual supervised PA group plus standard exercise counseling (PA + EC) versus a virtual supervised PA plus motivationally-enhanced behavioral counseling (PA + BC) group on moderate-to-vigorous intensity PA (MVPA) in people LWBC.

**Methods:**

This study is a two-armed, multi-site randomized controlled trial (RCT). People LWBC will be recruited and randomized to a 6-month virtual supervised PA intervention plus standard exercise counseling (PA + EC group; *n* = 118) or a 6-month virtual supervised PA plus behavioral counseling based on the Multi-Process Action Control (M-PAC) framework (PA + BC group; *n* = 118). Supervised PA will be delivered via synchronous Zoom classes that tapers to a home-based protocol at the end of the study. The goal of both groups is to gradually increase PA to the cancer PA guidelines (e.g., 90 min of MVPA/week). The PA + BC group will receive twelve behavioral counseling sessions with a qualified exercise professional (QEP), and the corresponding counseling session will be delivered bi-weekly. The behavioral counseling sessions will be based on the M-PAC’s reflective, regulatory, and reflexive processes. In addition to the supervised PA classes, the PA + EC (i.e., attention control group) will receive twelve standard PA counseling sessions based on PA training principles. People LWBC will complete measures at baseline, midpoint, post-intervention (6-months), at 6-months follow-up, and 1-year follow-up. Self-reported measures include quality of life (QoL), motivational outcomes, health economics, and patient satisfaction. Objective measures include PA via accelerometry. Multilevel modelling will examine change in the primary (i.e., PA) and secondary outcomes (i.e., motivational outcomes from the M-PAC, physical function, QoL) at the five time points.

**Discussion:**

This study will create greater understanding on efficacious programming to support PA maintenance that can be used by clinical and community-based organizations as a low-cost, supportive care tool to improve health outcomes for people LWBC.

**Trial registration:**

Clinicaltrials.gov ID NCT06624930.

## Background

Many people living with and beyond cancer (LWBC) suffer from long-term side effects, such as fatigue, depression, and muscle loss, which contribute to poor quality of life (QoL) [1–4]. Physical activity (PA) has a positive impact on clinical outcomes including improvements in overall QoL, reducing treatment-related toxicities and cancer-specific mortality [3, 5]. Despite these benefits, the majority of people LWBC are not meeting PA guidelines [6–8]. People LWBC are realizing that they can receive quality and engaging access to care that are live remote (i.e., real-time feedback) to self-manage their symptoms [9]. This represents a unique opportunity to test and deliver live remote PA interventions in both clinical supportive cancer care and research trials [10]. Short-term supervised PA programs can improve fitness and participant-reported outcomes in people LWBC [1–4], but PA declines significantly post-intervention and long-term adherence is often low [11–14]. To achieve long-term health benefits, behavior change must be sustained [15]. 

Recent cancer specific PA guidelines for people LWBC suggest a minimum of 90 min of moderate-to-vigorous intensity PA (MVPA) per week, and at least 2 days of large muscle strength training per week [8] to improve side effects of cancer treatment benefits [8, 16]. However, 74.8% and 86.1% of people LWBC are not currently meeting aerobic PA and combined PA guidelines (i.e., 150 min of MVPA/week and/or 2 days/week of resistance training), respectively [7]. Given that people LWBC face several barriers to engaging in in-person PA (e.g., distance from clinical/community programs, treatment-related side effects [17], there is a need to develop and assess the efficacy of live remote approaches [18, 19]. The quality and effectiveness of live remote interventions relative to non-telehealth home-based exercise or rehabilitation interventions are still unclear [20–22]. Theoretical approaches to identifying key motivational outcomes to facilitate the adoption and maintenance of PA are limited [15, 23–25]. Behavior change techniques such as self-monitoring, goal setting, social support, and action planning are shown to be effective techniques and produced the largest overall effect size for behavior change in people LWBC [14]. Behavior change interventions are complex with numerous interacting components that are often poorly described, especially with regard to how maintenance is defined [26]. This hinders the understanding of intervention components that might facilitate PA maintenance [15]. Behavior change interventions improve PA over the course of the intervention; however, PA declines are more pronounced as the length of time between follow-up assessments increase [27]. Nevertheless, the inclusion of theoretical components increases the likelihood of behavior change in these interventions [15, 28]. 

The dominant theoretical approach in PA and cancer survivorship studies are social cognitive theories [23, 24, 29]. While informative, these theories rarely focus on maintenance through enacting on intention-behavior gap mechanisms [30]. The Multi-Process Action Control (M-PAC) framework [30] has a causal structure where an individual moves from intention formation to adoption of action control and onto maintenance of action control. According to the M-PAC [30], *reflective processes* (i.e., instrumental attitudes [expected benefits from performing PA], affective judgements [expected pleasure from performing PA], perceived capability [one’s ability to perform PA] and perceived opportunity [perceived social/environmental circumstances to perform PA) are necessary for PA intention formation in people LWBC. When these expectations are strong and positive, they culminate in the formation of PA intention (i.e., decision to enact regular PA). The dominant determinant when beginning regular PA is marked by the enactment of regulatory processes. *Regulatory processes* represent the behavioral, cognitive, and affective regulation strategies (e.g., planning, monitoring, attention focus, emotion regulation) that are enacted to translate intention into PA. Finally, *reflexive processes* comprised of habit [learned cue-behavior associations] and identity [role self-categorization] are those constructs that develop as a consequence of repeated successful behavioral outcomes over time. Taken together, behavior change is the product of reflective, regulatory, and reflexive processes that have facilitated an initial intention into successful on-going behavior [30], including people LWBC [31–34].

Motivating people LWBC to be physically active requires behavioral skills training and understanding the behavioral mechanisms for change which our study addresses. In a critical narrative review [26], 20 theories and conceptual papers on PA maintenance were identified with the social cognitive (e.g., Social Cognitive Theory), humanistic (i.e., Self-determination Theory), and socioecological approaches (i.e., Ecological Model of Physical Activity) which have left maintenance undifferentiated from initiation. Kwasnicka et al.’s [35] review identified five themes (e.g., maintenance motives, self-regulation, habits) that may need to be considered for maintenance, and the associated constructs within these themes (e.g., dual process models and habits, identity). The M-PAC includes all of these themes outlined above and is predicated on multiple lines of behavioral research in PA [26]. Thus, there is strong empirical evidence for M-PAC in both behavioral prediction and change in general populations and people LWBC [27, 36, 37]. There is considerable evidence (> 40 studies) for the M-PAC structure and constructs in terms of predictive validity, as well as sensitivity of change of these measures in randomized controlled trials (RCT) [28, 30, 37, 38] in people LWBC [31–34]. Specifically, a pilot study demonstrated the feasibility of delivering the M-PAC constructs in prostate cancer survivors over 12 weeks [32]. The behavioral counseling sessions were based on the M-PAC, and included behavior change techniques such as social support, goal setting, self-monitoring, action planning, habit, and identity. The behavioral counseling group increased MVPA by 24 min more than the exercise counseling group did. Preliminary evidence suggests that adding behavioral counseling to supervised PA in people LWBC may be feasible and result in better adherence to PA compared to exercise counseling alone, although this needs to be replicated in larger trials with sufficient follow-up period (i.e., ≥ 6 months) for PA maintenance. The addition of reflexive processes (habit, identity) [26, 35], which have received less attention, may be critical constructs that explain PA maintenance [39–42]. Furthermore, factors influencing PA adherence and maintenance have largely ignored sex and gender differences (i.e., socially manufactured roles, behaviors, expressions, identities) [43]. 

Therefore, the primary purpose of this study is to compare the effects of delivering an entirely virtual 6-month supervised MVPA group plus standard exercise counseling (PA + EC) versus a virtual supervised MVPA plus motivationally-enhanced behavioral counseling (PA + BC) group on MVPA in people LWBC. We hypothesize that the PA + BC group will increase MVPA compared to the PA + EC group at mid-intervention (T1; 3 months), post-intervention (T2; 6 months), 6-month follow-up (T3; 6 months post-intervention), and 1-year follow-up (T4; 1-year follow-up from post-intervention). Secondary outcomes include: (1) to determine the effects and cost-effectiveness of PA + EC versus PA + BC on secondary outcomes, including changes in reflective, regulatory, and reflexive processes from the M-PAC, physical function, QoL and costs; (2) to examine the regulatory and reflexive mediators of the 6-month intervention on MVPA; and (3) to examine moderators of treatment efficacy in the PA intervention. We will compare across various demographics (e.g., intersection of age, sex, gender, ethnicity, body mass index), fitness (e.g., baseline PA), and clinical subgroups (e.g., cancer type), as well as adherence and intervention completion across groups. This study will address gaps in the PA maintenance literature to demonstrate: (1) changes in absolute values across behavioral performance of people LWBC, (2) an increase in their magnitude of effect on PA over time; [26] and (3) sex and gender differences in PA maintenance.

## Methods/design

Ethics approval was granted by the Research Ethics Board at the University of Toronto (protocol #44916). The study was registered with ClinicalTrials.gov (NCT06624930) on October 3, 2024 and the study protocol was developed using the SPIRIT (Standard Protocol Items: Recommendations for Interventional Trials) 2013 statement [44].

### Study design

**Fig. 1:**
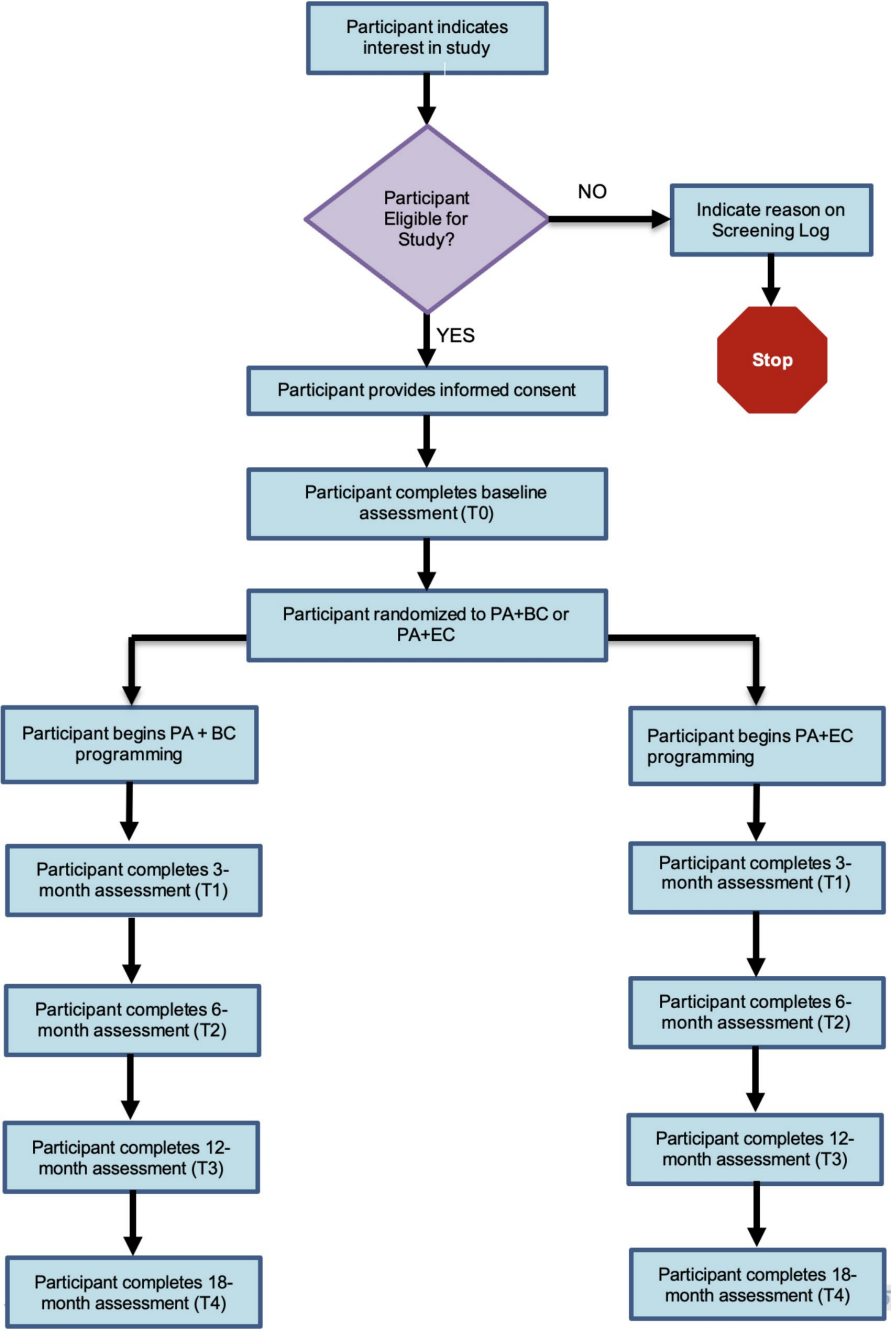
Study Flow Diagram

This study is a two-armed, multi-site RCT, to examine the effects of PA + EC versus PA + BC on MVPA in people LWBC. A 6-month follow-up (T3) and 1-year follow-up (T4; 1-year follow-up from post-intervention) will take place after the intervention to address maintenance. The study flow is outlined in Fig. 1 and the study measurements, outcomes, and schedule are summarized in Table 1.

**Table 1:** Outcome measures at each time point with time required for completion

Domain/Measure	Time Required for Completion	Baseline (month 0;T0)	Mid-point (month 3; T1)	Post-intervention (month 6; T2)	6-month follow-up (month 12; T3)	1-year follow-up (month 18; T4)
Eligibility		X				
Informed consent		X				
Group allocation (after completion of baseline, T0)		X				
**Interventions**						
*Intervention Group* - Physical Activity + Behavioral Counseling Group (PA + BC)						
*Attention Control Group* - Physical Activity + Exercise Counseling Group (PA + EC)						
**Measures**						
**Primary outcome**						
Physical activity (ActiGraph accelerometers)	Worn for 7 days during waking hours only	X	X	X	X	X
**Secondary outcomes**						
Quality of life (Functional Assessment of Cancer Therapy [FACT]-General, FACT-Fatigue, Fatigue subscale, EuroQol-5D)	20 min	X	X	X	X	X
Measures from the Multi-process Action Control (M-PAC) framework (affective attitudes, instrumental attitudes, injunctive norms, descriptive norms, and perceived control, behavioral regulation for physical activity, physical activity habits, exercise identity)	15 min	X	X	X	X	X
Physical function (i.e., 30-second chair stand, 6-minute walk test)	30 min	X	X	X	X	X
Demographic, medical and health history questionnaire	10 min	X				
Health economics (Health Services Utilization Inventory, iMTA Productivity Cost Questionnaire, Incremental Cost of Intervention)	20 min	X	X	X	X	X
Participant satisfaction questionnaire	5 min			X		
Adherence and compliance (Physical activity log)	5 min	assessed throughout intervention				
Fidelity and quality control (Fidelity checklists)	5 min	assessed throughout intervention				
Adverse events (Adverse events log)	5 min	assessed throughout intervention				

### Recruitment procedures

#### Participant eligibility

Eligibility for this study include those who are: 1) ≥ 18 years of age, 2) have a confirmed diagnosis of cancer of any type (Stages I to III; localized), 3) have completed primary cancer treatments within 5 years, 4) at least 12 weeks post-surgery and/or 6 weeks post-radiation, 5) are proficient in English, 6) are physically inactive (self-report < 90 min of MVPA/week) [45], 7) ambulate in daily life with minimal gait aid use (i.e., aid versus no aid), 8) have access to and can operate a smartphone/tablet/computer with a webcam for videoconferencing and a Bluetooth connection, 9) have access to the internet, and 10) have no cardiac contraindications (e.g., unstable angina, heart failure, coronary artery disease, diagnosed abnormality of heart rhythm). Participants are excluded if they: (1) have a medical condition that prohibits PA (e.g., joint restriction or weight bearing precautions), (2) have uncontrolled comorbidities or cardiovascular contraindications that would increase risk associated with supervised and unsupervised PA (e.g., cardiac contraindications, severe arthritis, recent fall within last 6–12 months), (3) advanced cancer (i.e., Stage IV; metastatic), and (4) do not intend to live in Canada for the next 18 months.

#### Participant recruitment

A total of 236 participants (*n* = 118 in each study group) will be recruited through listservs and flyers distributed in the community and posted in local cancer organizations. Recruitment will be performed through word of mouth and advertisements both online and through local community cancer support centers.

#### Screening procedure and informed consent

Potential participants will be screened by study staff over the phone to determine if they meet the eligibility criteria. Physician’s clearance for participation will be sought following PA guidelines outlined by American College of Sports Medicine (ACSM) and the National Comprehensive Cancer Network Survivorship Guideline [46, 47]. If the participant is eligible and willing to consent, study staff will review the informed consent process with the participant.

#### Randomization

Participants will be 1:1 randomized to either the PA + EC or the PA + BC group. Randomization will be performed using a stratified randomization scheme in the REDCap Randomization Module after baseline assessments. The sequence will be random permuted blocks of varying sizes, stratified by prior chemotherapy (yes versus no), age (< 65 versus ≥ 65 years), and sex (female versus male) to balance treatment characteristics that would potentially impact the severity of fatigue and intervention response [46]. Randomization codes will be kept in a pre-made digital file that is separate from other study records. Only the research co-ordinator will have access to the randomization codes. Following randomization, the research co-ordinator will update the randomization log to indicate the code has been used and include the ID number of the participant it was assigned to.

#### Blinding

All participants will be partially blinded to group assignment as they will not be told which study group will yield greater benefits as both groups receive supervised PA. The research co-ordinator will be responsible for scheduling all data collection assessments with research assistants (RAs) who are blind to group allocation. An independent group of RAs (separate than those who are engaged in data collection) will conduct the behavioral counseling and group webinars.

#### Sample size

The power calculation, based on a mixed model test with two intervention levels (i.e., PA + BC and PA + EC) and one dependent variable (i.e., MVPA), was performed in G*Power [48] to estimate the sample size to detect the global effect of treatments on outcomes. Based on our pilot trials [32, 49] and benchmarks for PA interventions in people LWBC [50], the total sample size of 196 will provide 80% power with alpha 0.05 to detect a difference of 24 min per day of MVPA between the groups based on pilot data [32]. Attrition rates are expected to be 20% [51], and thus the target sample size will be 236 (i.e., 118 in the PA + BC group and 118 in the PA + EC control group) to detect meaningful differences in MVPA. Equal representation of males, females, and other gender identities will be recruited to ensure sufficient power for sex and gender analyses.

#### Study groups

The goal of both groups is to gradually increase PA to a minimum of 90 min of MVPA per week following the updated ACSM PA guidelines for people LWBC [46]. People LWBC in both groups will be given an individualized, home-based aerobic and resistance prescription based on baseline assessment and previous work (Table 2) [32, 49]. The duration and intensity will account for the participant’s baseline physical function results, PA history, and preferences. The prescribed intensity will be 40–59% of the maximum heart rate reserve (i.e., moderate intensity) for weeks 1–6 and 60–70% (i.e., moderate to vigorous) by the end of the intervention. Initial PA duration will also be individualized but generally begin at 10–20 min per session, gradually increasing aerobic duration by 5 min each subsequent week. An aerobic duration of 30 min will be achieved by and maintained after week 6 of the intervention to meet PA guidelines [52]. Following aerobic PA, a standard progressive, resistance training program with resistance bands provided to each participant for two or three sets of 8–12 repetitions each at an intensity of 12–15 on a rating of perceived exertion (RPE; 6–20) scale (approximately 60–80% of one-repetition maximum). The resistance exercises will target all major muscle groups (e.g., seated row, chest press, squats, biceps curl). Heart rate monitors (i.e., Fitbits provided to each participant) will be used to encourage PA within target heart rate zones and for self-monitoring. These wearables have been incorporated into several interventions as an intervention tool to increase PA as they provide effective self-monitoring for PA intensity [53, 54]. 

**Table 2:** Description of the physical activity intervention

Week	1–2	3–4	5–6	7–8	9–10	11–12	13–18	19–24
Number of sessions/week	3	3	3	3	3	3	2	1
Rating of Perceived Exertion (RPE)	12–13 (aerobic) 12–15 (resistance)	12–13 (aerobic) 12–15 (resistance)	13–14 (aerobic) 12–15 (resistance)	13–14 (aerobic) 12–15 (resistance)	14–15 (aerobic) 12–15 (resistance)	14–15 (aerobic) 12–15 (resistance)	15–16 (aerobic) 12–15 (resistance)	15–16 (aerobic) 12–15 (resistance)
Intensity for aerobic physical activity (% Heart Rate Reserve)	40–59	40–59	50–59	50–59	55–65	55–65	60–70	60–70
Duration of physical activity (min)	10–15 (aerobic) 15–20 (resistance): 1 set of 8–12 reps for 60–80% of one-repetition maximum	15–20 (aerobic) 20–30 (resistance): 2 set of 8–12 reps for 60–80% of one-repetition maximum	15–20 (aerobic) 20–30 (resistance): 2 set of 8–12 reps for 60–80% of one-repetition maximum	20–25 (aerobic) 30 (resistance): 2–3 sets of 8–12 reps for 60–80% of one-repetition maximum	25–30 (aerobic) 30 (resistance): 2–3 sets of 8–12 reps for 60–80% of one-repetition maximum	25–30 (aerobic) 30 (resistance): 2–3 sets of 8–12 reps for 60–80% of one-repetition maximum	25–30 (aerobic) 30 (resistance): 2–3 sets of 8–12 reps for 60–80% of one-repetition maximum	25–30 (aerobic) 30 (resistance): 2–3 sets of 8–12 reps for 60–80% of one-repetition maximum

**Fig. 2:**
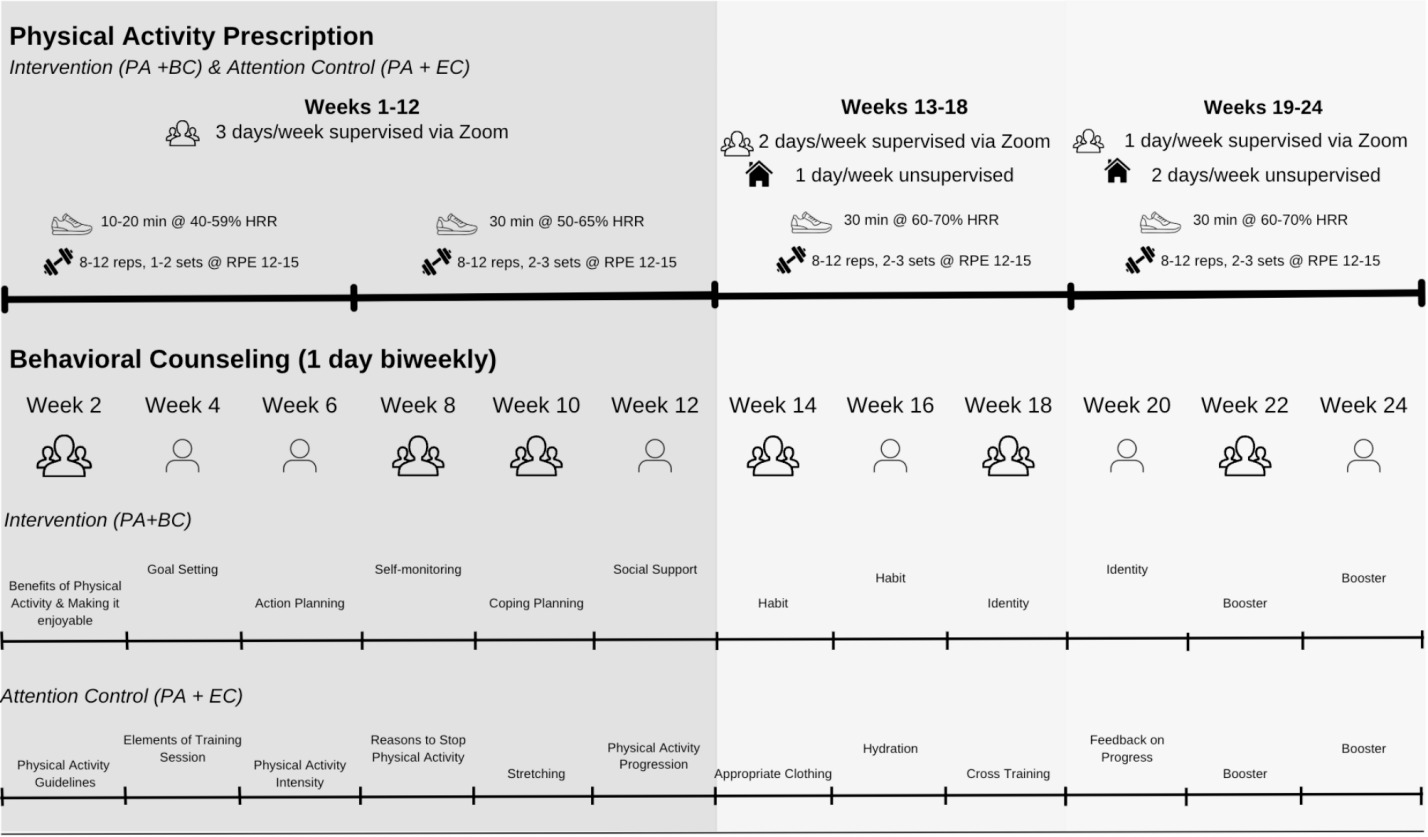
Overview of Study Components

Following all baseline testing delivered remotely (i.e., physical function), participants will be randomized to one of two groups: PA + BC or PA + EC (attention control). People LWBC in both groups will receive a tapered, home-based program lasting 1 hour for 3 days per week for the first 12 weeks, 2 days per week for week 13–18, and then 1 day per week for week 19–24 (54 sessions total). Qualified exercise professional (QEP)-led, group-based (~ 12 participants per class [55]) PA sessions completed at home via videoconferencing (i.e., Zoom), resistance bands, and an exercise mat will be provided for both groups. Consideration of the different cancer types are addressed through the tailored PA prescriptions and individual counseling sessions. Additional at home/unsupervised sessions will be prescribed, where people LWBC will be asked to complete one PA session on their own for weeks 13–18, and two PA sessions for weeks 19–24 (Fig. 2). Tracking and monitoring tools (i.e., Fitbits, PA logs) will be provided to ensure intensity and duration are met for both groups. A PA manual will be distributed to each participant as an ongoing resource, which will be developed separately for each of the two groups, with the behavioral counseling group containing more content (i.e., theory-based counseling). Sessions will be delivered by QEPs which encompasses graduate students in Kinesiology who are certified trainers with the Canadian Society of Exercise Physiologists (CSEP) or ACSM, as well as Registered Kinesiologists or Physiotherapists with experience working with people LWBC at the University of Toronto site for consistency. Training sessions/manuals will be provided for strict protocol adherence including core delivery components for the counseling sessions, cancer treatment information, PA safety and barriers, and quality control checklists.

*Intervention Group - Physical Activity Group + Behavioral Counseling Group (PA + BC)-*The PA + BC group will receive a behavioral counseling support session with a QEP every two weeks during the intervention period (12 total). The support sessions will be 30–45 min and delivered via Zoom each week in either group or 1:1 format depending on the topic. The videoconference calls will incorporate tailored behavior change content from the M-PAC framework (Table 3; Fig. 2). Of the 12 video-conferencing calls, one session will target reflective processes (instrumental/affective attitudes), five sessions will target behavioral regulation (action planning, coping planning, social support, goal setting, emotion regulation), and four sessions will target reflexive processing (identity, habit). The remaining two sessions are “booster sessions” to revisit topics discussed. The importance of sustaining PA for clinical outcomes (e.g., fatigue) and PA logs will be stressed. At the end of the 6-month intervention, an individualized PA prescription will be provided based on their fitness level (adjusted throughout) to continue achieving the PA goal for the 6-month and 1-year follow-up.

*Attention Control Group  Physical Activity Program + Exercise Counseling Group (PA + EC)-*The PA + EC group will receive the same frequency of group-based and 1:1 counseling support sessions via Zoom as PA + BC participants. However, the focus will be on PA training principles for proper PA technique, how to monitor intensity, and progress PA safely to achieve the PA guidelines. The support sessions will be 30–45 min and delivered via Zoom each week in either group or 1:1 format depending on the topic (Table 3; Fig. 2).

**Table 3:** Bi-Weekly videoconferencing topics for the supervised physical activity group plus behavioral counseling (PA + BC; intervention group) and supervised physical activity group plus exercise counseling (PA + EC; attention control group)

Week& Format	Description of Behavioral Strategies (PA + BC group)	Multi-process Action Control (M-PAC) Constructs for PA + BC Group Mapped to Behavior Change TechniquesUsed in Michie et al. (2013)^a^	Topics for Attention Control Group (PA + EC group)
2 Group	*Target M-PAC construct: Reflective processes (instrumental/affective attitudes)* Discuss the benefits of increasing physical activity for health and clinical outcomes, and how to make activity enjoyable	5.1 Information about health consequences 5.2 Salience of consequences 5.3 Information about social and environmental consequences 3.3 Social support (emotional) 5.6 Information about emotional consequences 7.5 Remove aversive stimulus 12.5 Adding objects to the environment	Physical activity guidelines
4 1:1	*Target M-PAC construct: Regulatory processes (goal setting)* Setting challenging, yet achievable goals using the SMART goal guidelines and developing rewards for achieving goals	11.1 Goal setting (behavior) 1.2 Problem solving 1.3 Goal setting (outcome) 1.8 Behavioral contract 2.3 Self-monitoring of behavior 2.6 Biofeedback 7.1 Prompt/cues 10.7 Self-incentive 11.2 Reduce negative emotions 13.4 Valued self-identity	Components of a physical activity training session
6 1:1	*Target M-PAC construct: Regulatory processes (action planning)* Action planning will be targeted by creating plans for increasing physical activity	1.1 Goal setting (behavior) 1.2 Problem solving 1.8 Behavioral contract 2.3 Self-monitoring of behavior 2.6 Biofeedback 7.1 Prompt/cues 10.7 Self-incentive 11.2. Reduce negative emotions	Physical activity intensity (e.g., heart rate training)
8 Group	*Target M-PAC construct: Regulatory processes* (self-monitoring) Discussion of how to monitor physical activity using wearables (e.g., Fitbits) and logs to keep track of progress towards goals	1.1 Goal setting (behavior) 1.2 Problem solving 1.4 Action planning 1.8 Behavioral contract 2.3 Self-monitoring of behavior 2.6 Biofeedback 7.1 Prompt/cues 10.7 Self-incentive 11.2 Reduce negative emotions 15.4 Self-talk	Signs and symptoms for stopping physical activity
10 Group	*Target M-PAC construct: Regulatory processes (coping planning*,* emotion regulation)* Discussing common psychologic, emotional, and environmental barriers to physical activity and strategies to overcome them	1.1 Goal setting (behavior) 1.2 Problem solving 1.4 Action planning 1.8 Behavioral contract 2.3 Self-monitoring of behavior 2.6 Biofeedback 7.1 Prompt/cues 10.7 Self-incentive 11.2 Reduce negative emotions 15.4 Self-talk	Stretching routine
12 1:1	*Target M-PAC construct: Regulatory processes (social support)* Discussion of social support and how to obtain support from significant others (i.e., spouse, friend, siblings)	3.2 Social support (practical) 12.1 Restructuring the physical environment 12.2 Restructuring the social environment	How to progress safely through a physical activity program
14 Group	*Target M-PAC construct: Reflexive processes (habit)* Identifying environmental cues to increase physical activity such as cues paired with time of the day, place, event, or other habits	7.1 Prompt/cues 7.5 Remove aversive stimulus 7.8 Associative learning 8.1 Behavioral practice 8.3 Habit formation	What to wear during physical activity
16 1:1	*Target M-PAC construct: Reflexive processes (habit)* Reinforcing a physical activity routine based on tagging physical activity to existing schedule and reminders strategies to reinforce the cues	7.1 Prompt/cues 7.5 Remove aversive stimulus 7.8 Associative learning 8.1 Behavioral practice 8.3 Habit formation	Importance of hydration
18 Group	*Target M-PAC construct: Reflexive processes (identity)* Building a physical activity identity by brainstorming and prioritizing various identities and where physical activity fits in	6.2 Social comparison 8.1 Behavioral practice 8.2 Behavioral substitution 12.5 Adding objects to the environment 13.2 Framing/reframing 13.3 Incompatible beliefs 13.4 Valued self-identity 13.5 Identity associated with changed behavior	Cross training
20 1:1	*Target M-PAC construct: Reflexive processes (identity)* Continue building a physical activity identity by prioritizing physical activity and finding personal meaning in participating in physical activity	6.2 Social comparison 8.1 Behavioral practice 8.2 Behavioral substitution 12.5 Adding objects to the environment 13.2 Framing/reframing 13.3 Incompatible beliefs 13.4 Valued self-identity 13.5 Identity associated with changed behavior	Feedback for progress to date
22 Group	*Booster session* Participants are free to raise any challenges and barriers to their activity goals or to revisit previous topics discussed	Behavior change techniques will depend on session revisited	Feedback for progress to date or revisit one of the earlier topics discussed
24 1:1	*Booster session* Participants are free to raise any challenges and barriers to their activity goals or to revisit previous topics discussed	Behavior change techniques will depend on session revisited	Feedback for progress to date or revisit one of the earlier topics discussed

### Data collection

All primary and secondary outcomes will be measured at baseline (T0), midpoint (i.e., 3 months into intervention; T1), T2 (i.e., immediately post-intervention [6 months]), T3 (i.e., 6-month follow-up from post-intervention), and T4 (i.e., 1-year follow-up from post-intervention).

#### Primary outcome

*Moderate-to-Vigorous Intensity Physical Activity* - Changes in MVPA will be assessed by accelerometry (ActiGraph Inc., Pensacola, FL.; model GT3X +). [56]Participants will be mailed an accelerometer to wear on their right hip, fastened to a belt worn around the waist. The accelerometer will be worn during waking hours for 7 days, except when bathing or swimming and returned to study staff via a postage-paid envelope. Activity data will be collected in one-minute intervals (epochs), with the total number of counts for each day summed and divided by the number of days of monitoring to arrive at an average number of activity counts. Cut-points will be based on Freedson et al. [56](i.e., 100–1,951 counts•minute − 1 for light PA; ≥1952 counts•minute − 1 for MVPA). The ActiLife© software will be used for processing and analysis of the data. The device is accurate for predicting energy expenditure (and time spent in PA) for walking (2–5 mph) and running on a flat surface (~ 5.6 mph; 0% slope [57]. The participant will also be mailed the accelerometer wear time log and the instructions to ensure the devices are worn properly and wear time is tracked.

#### Secondary outcomes

*Self-reported Physical Activity -* PA will be measured using a modified version of the (GLTEQ) Godin-Leisure Time Exercise Questionnaire [45] This measure asks participants to self-report the frequency and duration of light, moderate, vigorous intensity aerobic PA and resistance training for a typical week. This measure exhibits good reliability with coefficients of 0.83 and 0.85 [45]. 

*Reflective*,* Regulatory*,* and Reflexive Processes -* Standard measures from the M-PAC framework will be assessed including reflective processes of attitudes, and perceived capability and opportunity on a 7-point bipolar Likert scale [30, 31]. These items have been used with people LWBC [31, 32, 58]. Regulatory processes will be assessed with six items examining action and coping planning for PA using a 7-point scale (i.e., no plans–detailed plans) [59] Additionally, the Physical Activity Regulation Scale [59] be used as an additional measure of regulatory processes. The 14-item questionnaire measures proactive and reactive regulation, social monitoring, and self-monitoring and has displayed good internal consistency (> 0.80) [59]. Reflexive processes will be measured with the self-reported automaticity subscale that characterizes habit by automatic activation, behavioral frequency, and relevance to self-identity [60], and the exercise identity scale [61, 62] Cronbach’s alpha of these measures in previous studies in cancer range from 0.77 to 0.97 [62]. The M-PAC constructs have been validated and reliable based on years of prior research (see Table 3 in Rhodes et al., 2017 [30]paper).

*Physical Function -* Select measures from the Senior Fitness Test [63] will be used to assess physical function remotely: 30-s chair stand test for lower body strength and the 6-minute walk test for aerobic endurance. Higher scores or distance walked indicates better physical function across the domains.

*Quality of Life -* QoL will be assessed by the validated Functional Assessment of Cancer Therapy-General (FACT-G) which consists of physical well-being (PWB), functional well-being (FWB), emotional well-being (EWB), and social well-being (SWB) [64]. The FACT-Fatigue (FACT-F) scale includes the 27 items from the FACT-G scale plus the 13-item fatigue subscale, which is our primary QoL outcomes [64, 65]. Test-retest reliability is 0.92 for the total scale and 0.82–0.88 for subscales [64]. QoL will also be assessed with the EuroQol-5D (Eq. 5D) [66] as a part of the evaluation of cost-effectiveness.

*Demographic*,* Gender*,* Medical Information and Participant Satisfaction -* Demographic variables include: age, sex, gender, marital status, highest level of education, current employment status, ethnicity, PA history, and body mass index. Medical variables include: cancer type, time since diagnosis, disease stage, current/prior treatments, previous recurrence, current disease status, and comorbidities [67, 72]. Participant satisfaction items will assess burden and overall impressions similar to our prior work in people LWBC [32, 49]. We will disaggregate and analyze all primary and secondary outcomes by gender and sex [68]. 

Other demographic (e.g., age, ethnicity) and clinical (e.g., time since treatment) variables that intersect with gender will also be considered. The approach recommended by experts in gender-related measures is the two-step approach (Centre of Excellence for Transgender Health), which involves asking participants about both their current gender identity and their sex assigned at birth [69]. The Gender Index [70–72] will be used which includes all four aspects of gender (e.g., gender roles, gender relations, institutionalized gender, gender identity). Specific M-PAC constructs (e.g., perceived capability, habit, identity) may vary by sex and gender, thus targeting these considerations may maximize the potential effectiveness of PA interventions [41, 73]. This will prompt gender-specific, patient-centric care pathways for PA interventions designed to maximize the effectiveness of PA and patient outcomes.

*Health Economics Evaluation -* Health Economics Evaluation-Costs will be tracked using the Health Service Utilization Inventory [74] and the iMTA Productivity Cost Questionnaire [75] to conduct cost-effectiveness analyses. The incremental cost of the intervention will be estimated and compared to changes in utility values converted from EQ5D results [76] to evaluate the incremental cost per quality-adjusted life year (QALY) of the PA + BC group. This study will be conducted from the perspective of the Canadian public healthcare payer and the participant. This inventory will be partially completed by each participant as well as members of the research team. Participants will complete the Health-Service Utilization Inventory and the research team will complete the iMTA Productivity Cost Questionnaire.

#### Statistical analysis

A series of multilevel models using full information maximum likelihood to examine changes in the primary (i.e., MVPA) and secondary outcomes (i.e., reflective, regulatory, and reflexive processes from the M-PAC, physical function, QoL) will be conducted at the four time points. Multilevel modelling allows for simultaneous assessments of within person variations at each time measurement (level 1), which will be nested within study group (level 2). Finally, study group will be nested within study site (level 3). Although our primary time point of T2 will confirm the adherence reported post-intervention, inclusion of the T3 and T4 assessments allows us to determine if adherence is maintained. Fixed effects included in models will be time (i.e., baseline, T1, T2, T3, and T4), group (i.e., PA + BC intervention and PA + EC attention control), and interaction. All models will be estimated while adjusting for covariates (e.g., baseline PA, age, sex, treatment and cancer type). We will use the same analytical approach to assess differences across subgroups (e.g., sex, gender, drop-outs) by modeling the changes over time. Differences between men and women will be analyzed by adding a gender indicator variable to each of the models. The gender covariate will be crossed with the model’s independent variable so their interaction will be in the model. An intention-to-treat analysis will be used. For missing data at post-intervention or follow-up, all available data will be included under the missing-at-random assumption of the mixed-model analysis. Additionally, sensitivity analyses will be conducted to evaluate the robustness of our findings under alternative missing data assumptions, such as not-missing-at-random. The PROCESS macro for SPSS [77] will be used to examine mediation effects of the reflective, regulatory, and reflexive processes between groups (PA + BC group; PA + EC group) and MVPA and indirect effects across the path model. Cost and QALY differences between groups will be calculated using ordinary least squares regression analyses. Incremental cost-effectiveness ratios (ICER) will be calculated by dividing the adjusted incremental costs by incremental effects. ICER uncertainty will be measured by bootstrapping using at least 5000 replications.

#### Monitoring

All personnel will be trained at the University of Toronto site to ensure quality control and fidelity (e.g., manual of operations, provider training [3 × 6-hour sessions], weekly conference calls with sites, intervention delivery checklists) using guidelines recommended by the National Institutes of Health (NIH) Behavior Change Consortium [78]. This includes best practice recommendations to address treatment fidelity across five domains: Study Design (e.g., user-friend scripted counseling sessions), Provider Training (e.g., role plays for skill acquisition), Treatment Delivery (e.g., ensure adherence to protocol via checklists), Treatment Receipt (e.g., assess patient’s confidence to apply skills learned), and Treatment Enactment (e.g., self-report engaging in physical activity). QEPs will undergo role plays with a scoring guide to ensure standardization.

A data audit will take place at least once per year to ensure forms are being completely accurately and timely. Depending on the pace of recruitment, these data audits may take place more frequently. For safety monitoring, all adverse events will be classified as either an adverse (AE) or a serious adverse event (SAE). These events may occur during screening and baseline data collection, or they may occur during the administration of the intervention. An adverse event may or may not be related to the data collection or intervention procedures. When an adverse event is identified, study personnel identifying the adverse event will report it to the research s and the Principal Investigator. The research co-ordinator will then contact the participant and an electronic Adverse Event Report Form will be submitted to the research ethics board will be completed within 7 days of the event and within 48 h for serious adverse events. Decisions to discontinue or modify the intervention for a participant will be on a case-by-case basis by the research team.

## Discussion

This study will test a theory-based, distance-based intervention that is accessible, eliminates barriers, and reaches large numbers of people LWBC to integrate behavioral counseling in live remote options in communities and clinical care. In our pilot study, we consulted people LWBC (i.e., target users) in developing the intervention to consider their needs and preferences for a PA intervention [62]. We then tailored the intervention and further solicited feedback from their experiences [32, 49] to refine this current study. While our pilot study demonstrated high satisfaction with the intervention components, feedback was provided on making the counseling sessions more engaging which may result in greater uptake of behavior change techniques. Consequently, in this current study, the supervised in-person PA sessions were replaced with a live remote, group-based PA classes and 1:1 videoconferencing with a QEP. PA preferences in people LWBC suggest strong preferences towards home-based programs [79], the use of digital or remote platforms (e.g., Zoom, other online and video platforms) [10, 80], and the importance of social support [79]. Furthermore, our prior work did not include a PA program of sufficient duration and follow-up period. Therefore, the frequency of delivery of the behavioral support sessions were revisited as certain behavior change techniques such as prompts/cues and action and coping planning may need to be repeated for sustained PA. The current study includes group and individual counseling over a longer period (i.e., 6 months) with several booster sessions to revisit prior topics.

The findings will provide additional support for oncology clinicians to assess, advise, and refer patients to PA programs [19]. People LWBC have a strong desire for advice/support with healthy lifestyle programs [9, 81], which is not routinely offered as part of survivorship care. Overall, the pilot study will move from a feasibility to an efficacy study, focus on maintenance grounded in theory (e.g., M-PAC), include wearables (i.e., Fitbit) for self-monitoring, provide key M-PAC behavior change techniques, and identify best practices in live remote PA interventions. This study has high potential for broad reach and impact on most people LWBC by building a unique virtual care platform. In turn, this could be implemented in clinical and community-based organizations as a supportive care tool to increase PA that will ultimately improve health and fitness-related outcomes long-term for people LWBC.

## Data Availability

No datasets were generated or analysed during the current study.

## Abbreviations

ACSM: American College of Sports Medicine
AE: Adverse Event
BMI: Body Mass Index
CSEP: Canadian Society of Exercise Physiologists
EWB: Emotional well-being
EQ5D: EuroQol-5D
FACT-G: Functional Assessment of Cancer Therapy-General
FACT-F: Functional Assessment of Cancer Therapy-Fatigue
FWB: Functional well-being
GLETQ: Godin Leisure-time Exercise Questionnaire
ICER: Incremental cost-effectiveness ratio
KT: Knowledge translation
LWBC: Living with and beyond cancer
MVPA: Moderate-to-vigorous intensity physical activity
M-PAC: Multi-Process Action Control Framework
NIH: National Institutes of Health
PA: Physical Activity
PA + BC: Physical Activity + Behavioral Counseling
PA + EC: Physical Activity + Exercise Counseling
PAR-Q+: Physical Activity Readiness Questionnaire Plus
PWB: Physical well-being
QALY: Quality-adjusted life years
QoL: Quality of Life
QEP: Qualified Exercise Professional
RA: Research Assistant
RCT: Randomized Controlled Trial
REDCap: Research Electronic Data Capture
RKins: Registered Kinesiologists
RPE: Rate Perceived Exertion
SAE: Serious adverse event
SWB: Social well-being
